# Urban-rural differences in the prevalence of having a family dentist and their association with income inequality among Japanese individuals: a cross-sectional study

**DOI:** 10.1186/s12903-024-04528-8

**Published:** 2024-06-27

**Authors:** Katsuo Oshima, Hiroko Miura, Rumi Tano, Hideki Fukuda

**Affiliations:** 1 Department of Dental Technology, The Nippon Dental University College at Tokyo, 2-3-16 Fujimi, Chiyoda-ku, Tokyo, 102-0071 Japan; 2 Division of Disease Control and Epidemiology, School of Dentistry, Health Sciences University of Hokkaido, 1757 Kanazawa, Tobetsu-cho, Ishikari-gun, Hokkaido, 061-0293 Japan; 3 Department of Health Promotion, National Institute of Public Health, 2-3-6 Minami, Wako- shi, Saitama, 351-0197 Japan; 4 National Institute of Public Health, 2-3-6 Minami, Wako-shi, Saitama, 351-0197 Japan

**Keywords:** Family dentist, Urban-rural differences, Income inequality, Socioeconomic factors, Japan

## Abstract

**Background:**

Few reported studies evaluate the status of those who have a family dentist (FD) by regional differences and the socioeconomic factors associated with this status. This study aimed to assess the prevalence of having an FD among Japanese individuals across three samples of municipality type: urban, intermediate, and rural areas, and determine the factors associated with having an FD.

**Methods:**

This was a cross-sectional study involving a web-based survey. In total, 2,429 participants (comprising men and women aged 20–69 years) were randomly selected from among the registrants of a web research company: 811 urban residents, 812 intermediate residents, and 806 rural residents. In each area, we categorized the participants into those who had an FD (FD group) and those who did not (non-FD group). A multivariate modified Poisson regression analysis was used to determine the factors associated with the FD group as compared to the non-FD group.

**Results:**

The proportion of the FD group was lowest in rural areas (42.3%), followed by intermediate (48.6%) and urban areas (49.7%). The regression analysis revealed a statistically significant tendency between associated factors in the two groups; that is, the higher the household income, the more likely that the family belonged to the FD group (prevalence ratio (95%CI), JPY 4–6 million: 1.43 (1.00–2.03), JPY ≥ 8 million: 1.72 (1.21–2.44)).

**Conclusions:**

Rural areas have the lowest proportion of people with an FD among the three areas, and income inequality is associated with having an FD. Thus, when planning policies to encourage individuals to have an FD to manage their oral health, it is necessary to consider regional differences.

**Supplementary Information:**

The online version contains supplementary material available at 10.1186/s12903-024-04528-8.

## Background

Dental healthcare systems vary worldwide; however, in many countries, it is a commonly accepted fact that having a family dentist (FD) is positively associated with maintaining good oral health [1–5]. Previous studies have demonstrated that having an FD is linked to tooth retention [1, 2], higher satisfaction with oral healthcare [3] and trust in dentistry [4], and habitual oral health behaviors, such as teeth brushing and interdental cleaning [5].

In the Japanese dental healthcare system, although there is an understanding of the idea that having an FD can protect oral health through regular dental visits, it is not institutionalized, and having an FD is a personal choice. When patients receive dental care, most treatments are covered by public healthcare insurance, except for certain prosthetic treatments, and they pay a co-payment of 10–30%, depending on their age [6]. Patients are free to choose any dental clinic, regardless of the municipality in which they live and/or their public healthcare insurance type. As 97.7% of dental clinics in Japan (67,874 facilities as of 2020) advocate general dentistry [7], most Japanese dental clinics can be considered to have primary dental care functions.

Considering Japan’s dental healthcare system, a higher proportion of Japanese people would be expected to visit their dentists regularly and manage their oral health conditions; however, the proportion of those who practice such behavior is not high [8], which is one of the issues in Japanese health policy. Health Japan 21 (the third term), formulated by the Japanese Ministry of Health, Labour and Welfare in 2023, sets a target value of 95% by 2032 for the proportion of people who receive yearly dental check-ups (current value: 58% in 2022) [8]. The Japan Dental Association encourages Japanese people to have an FD who provides dental care and/or management continuously and is always available for consultation to protect their oral health throughout their lives [9].

In a nationwide survey conducted in Japan, the definition of having an FD was set (described in “Outcome variable” in Methods), and it was reported that 50.0% of study participants had an FD [5]. Additionally, previous studies have suggested that having an FD is associated with geographic accessibility to dental care (for women only) [10] and socioeconomic factors such as older age and higher income [5]. However, few studies have evaluated regional differences in the proportion of people with an FD, such as those between urban and rural areas, and the factors associated with these differences. Several previous studies have reported that regional differences influence residents’ receipt of dental care services [11–14]. By sampling an equal proportion of participants from each region using a web-based survey methodology, it is possible to compare each area. Understanding the actual status of individuals with an FD in each region is expected to provide meaningful data for the planning of oral health policies according to the characteristics of each region.

We established three samples of municipality type: urban, intermediate, and rural residents. This study aims to compare the proportion of people with an FD in each of these three areas and determine the factors associated with having an FD in each area.

## Methods

### Study design, setting, and participants

This cross-sectional study utilized a web-based survey that was conducted in Japan between November 24 and 28, 2022, by a research company (Macromill, Inc.) as part of a study by the Ministry of Health, Labour and Welfare. Approximately 1.3 million people are registered with Macromill, which is equivalent to 1% of the Japanese population [15].

The study participants were randomly selected from the research company’s registrants based on their municipality of residence, gender, and age. First, three groups were created by dividing municipalities into (1) urban areas (ordinance-designated cities with a population of 500,000 or more and 23 special wards of Tokyo), (2) intermediate areas (cities with a population of 100,000 or more, excluding urban areas), and (3) rural areas (cities with a population of less than 100,000, and towns and villages). Subsequently, in each group, quota sampling was conducted to ensure equal proportions for gender (men and women) and age (20s, 30s, 40s, 50s, and 60s); men and women aged 20–69 years were extracted. Considering the population of those aged 20–69 in each area (urban: approximately 24 million, intermediate: approximately 31 million, and rural: approximately 22 million) [13], we set a 50% population proportion, 95% confidence level, and the margin of error at 3.5%, which is as small as possible within the available study budget; this resulted in a target sample size of 800 participants in each municipality type. Ultimately, 2,429 study participants were included, comprising 811 from urban areas, 812 from intermediate areas, and 806 from rural areas.

The recruited participants were registered with the research company and provided their consent to participate in the study. The participants’ personal information was protected by the research company. This study was approved by the Ethics Committee of the Faculty of Dentistry, Health Sciences University of Hokkaido (October 2022; #232).

### Outcome variable

The criteria for the outcome variable of having an FD were [5, 9]: “Having a dentist with whom you can consult regarding any problem and who will refer you to a specialist, if necessary, and having received at least one dental check-up within the past year.” The participants were binarized into the FD and non-FD groups based on these criteria.

### Explanatory variables

The explanatory variables included gender (men/women), age (20–29, 30–39, 40–49, 50–59, and 60–69 years), household income (JPY < 2 million, JPY 2–4 million, JPY 4–6 million, JPY 6–8 million, JPY ≥ 8 million, and unknown), working status (regular worker, homemaker, part-time worker, and unemployed or others), number of teeth (≥ 28, 20–27, and ≤ 19), frequency of brushing teeth (≥ three times daily, twice daily, once daily, and sometimes or no brushing), and habitual interdental cleaning (yes/no).

All variables were treated as categorical. All items were mandatory to answer in order to submit the survey online; therefore, there were no missing data.

### Statistical analysis

The descriptive statistics for each variable were first evaluated. The proportion of the FD group for the urban, intermediate, and rural areas was then calculated and compared using the chi-square test.

Owing to the high prevalence of individuals with an FD, prevalence ratios (PR) and 95% confidence intervals (95%CI) were calculated using modified Poisson regression analysis with robust error variance (univariate analysis and multivariate analysis with the forced entry method), and the factors associated with having an FD (FD group = 1, non-FD group = 0) were evaluated [16]. Using a modified Poisson regression model, all survey participants were analyzed before stratifying by the three municipality types, and then analyzed by stratifying into each area type.

As supplementary analyses, FD status (FD or non-FD groups) and participant characteristics were set as explanatory variables, and each oral status (tooth number, frequency of brushing teeth, and interdental cleaning) was set as an outcome variable, then stratified by age groups. Univariate and multivariate analyses were performed for each oral status. The tooth number and frequency of brushing teeth were analyzed using negative binomial regression analysis, and interdental cleaning was analyzed using modified Poisson regression analysis.

Stata version 18 (StataCorp LLC, College Station, TX, USA) was employed for statistical analyses. Statistical significance was set at *p* < 0.05.

## Results

### Demographic characteristics of the study participants and breakdown of FD and non-FD groups, classified by municipality type

Table 1 shows the demographic characteristics of the study participants and the proportions of participants in the FD and non-FD groups in each of the areas. The participants analyzed in this study included 811 (50.2% men, 49.8% women) in urban areas, 812 (50.2% men, 49.8% women) in intermediate areas, and 806 (49.6% men, 50.4% women) in rural areas.

**Table 1:** Demographic characteristics of the study participants and breakdown of FD and non-FD groups, classified by municipality type

	Urban				Intermediate				Rural		
	Total	FD group	non-FD group		Total	FD group	non-FD group		Total	FD group	non-FD group
	n	(%)	(%)		n	(%)	(%)		n	(%)	(%)
Total	811	(49.7)	(50.3)		812	(48.6)	(51.4)		806	(42.3)	(57.7)
Gender											
Men	407	(48.9)	(51.1)		408	(43.4)	(56.6)		400	(36.0)	(64.0)
Women	404	(50.5)	(49.5)		404	(54.0)	(46.0)		406	(48.5)	(51.5)
Age (years)											
20–29	161	(41.6)	(58.4)		165	(35.8)	(64.2)		161	(38.5)	(61.5)
30–39	164	(44.5)	(55.5)		162	(51.9)	(48.1)		161	(41.0)	(59.0)
40–49	163	(49.1)	(50.9)		161	(41.6)	(58.4)		163	(40.5)	(59.5)
50–59	164	(47.6)	(52.4)		160	(56.9)	(43.1)		159	(42.1)	(57.9)
60–69	159	(66.0)	(34.0)		164	(57.3)	(42.7)		162	(49.4)	(50.6)
Household income (million)											
JPY < 2	69	(44.9)	(55.1)		75	(33.3)	(66.7)		93	(30.1)	(69.9)
JPY 2–4	143	(43.4)	(56.6)		145	(53.8)	(46.2)		164	(43.3)	(56.7)
JPY 4–6	162	(47.5)	(52.5)		153	(47.7)	(52.3)		156	(46.2)	(53.8)
JPY 6–8	115	(56.5)	(43.5)		133	(51.9)	(48.1)		112	(44.6)	(55.4)
JPY ≥ 8	173	(58.4)	(41.6)		133	(60.2)	(39.8)		98	(55.1)	(44.9)
Unknown	149	(45.0)	(55.0)		173	(40.5)	(59.5)		183	(36.1)	(63.9)
Working status											
Regular worker	466	(49.1)	(50.9)		417	(48.4)	(51.6)		406	(40.4)	(59.6)
Homemaker	109	(58.7)	(41.3)		133	(60.2)	(39.8)		122	(56.6)	(43.4)
Part-time worker	122	(47.5)	(52.5)		135	(51.1)	(48.9)		151	(45.0)	(55.0)
Unemployed/others	114	(45.6)	(54.4)		127	(34.6)	(65.4)		127	(31.5)	(68.5)
Number of teeth											
≥ 28	480	(44.8)	(55.2)		453	(47.5)	(52.5)		470	(40.2)	(59.8)
20–27	272	(58.8)	(41.2)		300	(51.3)	(48.7)		269	(44.6)	(55.4)
≤ 19	59	(47.5)	(52.5)		59	(44.1)	(55.9)		67	(47.8)	(52.2)
Frequency of brushing teeth											
≥Three times daily	231	(55.8)	(44.2)		221	(51.6)	(48.4)		231	(52.4)	(47.6)
Twice daily	424	(50.2)	(49.8)		428	(52.8)	(47.2)		410	(43.9)	(56.1)
Once daily	131	(39.7)	(60.3)		140	(39.3)	(60.7)		142	(26.1)	(73.9)
Sometimes/No brushing	25	(36.0)	(64.0)		23	(0.0)	(100.0)		23	(13.0)	(87.0)
Interdental cleaning											
Yes	472	(64.8)	(35.2)		423	(62.9)	(37.1)		420	(60.7)	(39.3)
No	339	(28.6)	(71.4)		389	(33.2)	(66.8)		386	(22.3)	(77.7)

Note: FD group = group of those who regularly manage their oral health with a family dentist (FD)

**Fig. 1:**
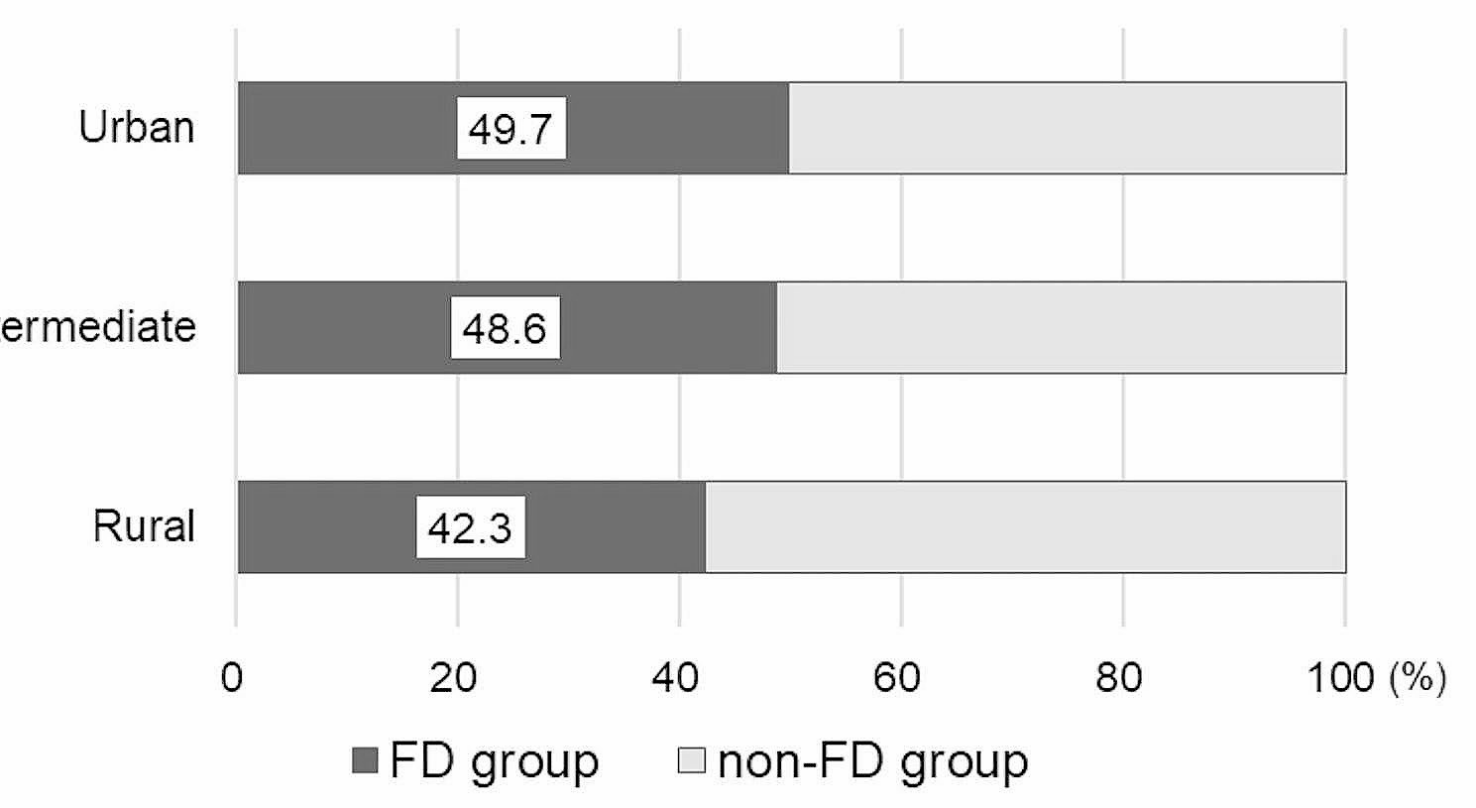
Proportion of the FD group in each municipality type Note: FD group = group of those who regularly manage their oral health with a family dentist (FD)

Figure 1 compares the proportions of the FD group for each of the areas. The proportions of the FD group were 49.7%, 48.6%, and 42.3% in the urban, intermediate, and rural areas, respectively. A chi-square test found a statistically significant difference between the three areas (χ^2^(2) = 10.4, *p* = 0.006). The FD group comprised 46.9% of all study participants (*n* = 2,429).

### Characteristics of the study participants in the FD group compared to the non-FD group, classified by municipality type

Table 2 shows the prevalence ratio (PR) and 95%CI calculated using modified Poisson regression analysis (multivariate analyses) for the associations between the FD group (FD group = 1, non-FD group = 0) and each explanatory variable. The results of the analysis for all participants (univariate and multivariate) without stratification by municipality type are presented in Supplementary Table 1 in the Additional file, and the results of the univariate analysis with stratification by municipality type are presented in Supplementary Table 2.

**Table 2:** Characteristics of the study participants in the FD group compared to the non-FD group, including all study participants and classified by municipality type (multivariate modified Poisson regression analysis)

	All study participants		Urban		Intermediate		Rural
	PR (95%CI)		PR (95%CI)		PR (95%CI)		PR (95%CI)
Gender							
Men	0.97 (0.88–1.08)		1.06 (0.91–1.25)		0.91 (0.77–1.08)		0.93 (0.76–1.14)
Women	reference		reference		reference		reference
Age (years)							
20–29	reference		reference		reference		reference
30–39	1.00 (0.87–1.15)		0.93 (0.73–1.18)		1.21 (0.94–1.54)		0.92 (0.71–1.18)
40–49	0.94 (0.81–1.08)		0.97 (0.77–1.22)		0.96 (0.74–1.25)		0.93 (0.72–1.19)
50–59	0.99 (0.86–1.14)		0.91 (0.72–1.16)		1.26 (0.98–1.62)		0.86 (0.68–1.10)
60–69	1.21 (1.05–1.39) **		1.29 (1.04–1.60) *		1.38 (1.07–1.78) *		1.03 (0.81–1.31)
Household income (million)							
JPY < 2	reference		reference		reference		reference
JPY 2–4	1.19 (0.99–1.44)		0.89 (0.66–1.19)		1.35 (0.96–1.91)		1.39 (0.98–1.96)
JPY 4–6	1.18 (0.97–1.43)		0.99 (0.74–1.32)		1.18 (0.82–1.70)		1.43 (1.00–2.03) *
JPY 6–8	1.28 (1.05–1.55) *		1.14 (0.85–1.52)		1.30 (0.91–1.86)		1.34 (0.93–1.92)
JPY ≥ 8	1.47 (1.19–1.75) ***		1.23 (0.93–1.63)		1.46 (1.03–2.07) *		1.72 (1.21–2.44) **
Unknown	1.08 (0.89–1.31)		0.96 (0.71–1.28)		1.09 (0.76–1.56)		1.28 (0.90–1.81)
Working status							
Regular worker	reference		reference		reference		reference
Homemaker	1.07 (0.94–1.21)		0.99 (0.80–1.22)		1.03 (0.83–1.27)		1.13 (0.90–1.43)
Part–time worker	1.03 (0.91–1.17)		1.01 (0.81–1.26)		1.02 (0.83–1.26)		1.03 (0.83–1.29)
Unemployed/others	0.92 (0.80–1.07)		0.99 (0.80–1.22)		0.86 (0.67–1.12)		0.91 (0.69–1.20)
Number of teeth							
≥ 28	0.90 (0.77–1.05)		0.96 (0.74–1.24)		1.02 (0.77–1.34)		0.73 (0.56–0.96) *
20–27	0.95 (0.82–1.11)		1.08 (0.84–1.39)		1.00 (0.76–1.32)		0.74 (0.56–0.98) *
≤ 19	reference		reference		reference		reference
Frequency of brushing teeth							
≥Three times daily	1.22 (1.06–1.41) **		1.10 (0.88–1.39)		1.15 (0.91–1.45)		1.48 (1.12–1.96) **
Twice daily	1.18 (1.03–1.34) *		1.06 (0.85–1.32)		1.18 (0.95–1.46)		1.37 (1.04–1.80) *
Once daily	reference		reference		reference		reference
Sometimes/No brushing	0.56 (0.33–0.95) *		0.95 (0.57–1.58)		0.00 (0.00–0.00)***		0.51 (0.16–1.66)
Interdental cleaning							
Yes	2.05 (1.85–2.28) ***		2.18 (1.81–2.62) ***		1.63 (1.39–1.91) ***		2.49 (2.02–3.06) ***
No	reference		reference		reference		reference
Municipality type							
Urban	1.10 (1.00–1.21)						
Intermediate	1.13 (1.02–1.25) *						
Rural	reference						

Note: * *p* < 0.05, ** *p* < 0.01, *** *p* < 0.001, FD group = group of those who regularly manage their oral health with a family dentist (FD); PR = prevalence ratio; 95%CI = 95% confidence interval

After adjusting for all explanatory variables, the multivariable analysis found statistically significantly higher PR in urban areas among those aged 60–69 years (PR (95%CI) = 1.29 (1.04–1.60)), and in those with interdental cleaning practices (2.18 (1.81–2.62)) (Wald χ^2^(19) = 130.0, *p* < 0.001). In the intermediate area, the PR was statistically significantly higher among those aged 60–69 years (1.38 (1.07–1.78)), those with higher household income (JPY ≥ 8 million: 1.46 (1.03–2.07)), and those with interdental cleaning practices (1.63 (1.39–1.91)) (Wald χ^2^(19) = 5696.3, *p* < 0.001). In rural areas, the PR was statistically significantly higher for those with higher household incomes (JPY 4–6 million: 1.43 (1.00–2.03), JPY ≥ 8 million: 1.72 (1.21–2.44)), those who practiced teeth brushing (≥ three times daily: 1.48 (1.12–1.96), twice daily: 1.37 (1.04–1.80)), and those with interdental cleaning practices (2.49 (2.02–3.06)); however, it was lower among those with more teeth (≥ 28: 0.73 (0.56–0.96), 20–27: 0.74 (0.56–0.98)) (Wald χ^2^(19) = 151.5, *p* < 0.001).

In Tables 3 and Supplementary Tables 3–5, the results of complementary univariate and multivariate analyses are presented, with FD status and participant characteristics as explanatory variables and each oral condition (tooth number, frequency of brushing teeth, and interdental cleaning) as outcome variables. With regard to the analysis in which the outcome variable was tooth number, there was no statistically significant association with FD status (Tables 3 and Supplementary Table 3). With regard to the analysis in which the outcome variable was the frequency of brushing teeth, there was a statistically significant association with the FD status in the analysis for all participants, but not for each age group (Tables 3 and Supplementary Table 4). As regards the analysis in which the outcome variable was interdental cleaning, there was a statistically significant association with FD status for all age groups (Tables 3 and Supplementary Table 5).

**Table 3:** Association between each oral status (tooth number, frequency of brushing teeth, and interdental cleaning) and each variable

	Tooth number		Frequency of brushing teeth		Interdental cleaning
	PR (95%CI)		PR (95%CI)		PR (95%CI)
Gender					
Men	0.98 (0.96–1.00)		0.87 (0.81–0.93) ***		0.77 (0.71–0.84) ***
Women	reference		reference		reference
Age (years)					
20–29	reference		reference		reference
30–39	1.00 (0.97–1.03)		1.03 (0.94–1.12)		1.29 (1.13–1.47) ***
40–49	1.00 (0.97–1.03)		1.02 (0.93–1.11)		1.34 (1.17–1.52) ***
50–59	0.95 (0.92–0.98) **		1.04 (0.95–1.14)		1.46 (1.29–1.66) ***
60–69	0.87 (0.84–0.90) ***		1.02 (0.93–1.12)		1.39 (1.22–1.57) ***
Household income (million)					
JPY < 2	reference		reference		reference
JPY 2–4	1.00 (0.96–1.04)		1.01 (0.90–1.14)		1.07 (0.92–1.25)
JPY 4–6	1.02 (0.98–1.06)		1.04 (0.92–1.17)		1.16 (1.00–1.36)
JPY 6–8	1.03 (0.99–1.08)		1.03 (0.91–1.17)		1.17 (1.00–1.37)
JPY ≥ 8	1.04 (0.99–1.08)		1.07 (0.94–1.20)		1.09 (0.93–1.27)
Unknown	1.03 (0.99–1.06)		1.02 (0.91–1.14)		1.00 (0.85–1.17)
Working status					
Regular worker	reference		reference		reference
Homemaker	1.02 (0.98–1.05)		0.97 (0.89–1.07)		1.03 (0.94–1.14)
Part–time worker	0.98 (0.95–1.01)		0.97 (0.89–1.05)		0.90 (0.81–1.01)
Unemployed/others	1.01 (0.98–1.04)		0.94 (0.86–1.03)		0.96 (0.85–1.09)
Municipality type					
Urban	1.00 (0.98–1.02)		0.99 (0.93–1.06)		1.06 (0.98–1.15)
Intermediate	1.00 (0.97–1.02)		0.99 (0.92–1.06)		0.96 (0.88–1.04)
Rural	reference		reference		reference
FD					
FD groups	1.01 (0.99–1.03)		1.09 (1.03–1.16) **		1.81 (1.67–1.96) ***
Non-FD groups	reference		reference		reference

Note: * *p* < 0.05, ** *p* < 0.01, *** *p* < 0.001, FD group = group of those who regularly manage their oral health with a family dentist (FD); PR = prevalence ratio; 95%CI = 95% confidence interval. The tooth number and frequency of brushing teeth were analyzed by performing multivariate negative binomial regression analysis, and interdental cleaning was analyzed by performing multivariate modified Poisson regression analysis

## Discussion

This study highlighted the following points regarding regional differences in the status of those with FD in Japan: (1) The proportion of those with an FD was lowest in rural areas (42.3%), followed by intermediate areas (48.6%) and urban areas (49.7%), (2) participants with higher household incomes in the intermediate and rural areas had a higher prevalence of having an FD.

Regarding urban-rural differences in dental health services, previous studies have focused on the utilization of preventive dental care services [11, 12], patient satisfaction with dental care [3], and oral health-related quality of life [17], all of which indicate that there are more barriers in rural areas than in urban areas. This study sampled equal proportions of participants in urban, intermediate, and rural areas, and found that the proportion of those who had an FD in each area was lower in the urban, intermediate, and rural areas, which supports the findings of past studies [3, 11, 12, 17]. A previous study found a 50% prevalence of having an FD among participants using a nationwide web-based survey in Japan which utilized a sampling method based on the Japanese census [5]. The current study sampled an equal-sized proportion of three areas based on municipality type, allowing for a larger proportion of participants from rural areas compared to the previous study; the proportion of those with an FD in urban and intermediate areas in the current study approximates that of the previous study [5].

According to the results of a multivariate modified Poisson regression analysis designed to evaluate the factors associated with having an FD regarding oral health status, in all three areas, those who habitually practiced interdental cleaning had a higher prevalence of having an FD (Table 2). Furthermore, as a supplementary analysis, a modified Poisson regression analysis with FD status as the explanatory variable and interdental cleaning as the outcome variable also showed a statistically significant association between the two (Table 3). Previous studies have shown that individuals who regularly manage their oral health through dental visits are positively associated with the practicing of habitual interdental cleaning [5, 18]. Interdental cleaning is an important behavior that protects oral health [19], and people who are habituated to such health behavior may have a positive perception of having an FD. Similarly, it is possible that the effect of having an FD leads to the practice of interdental cleaning; although this study is cross-sectional in nature and thus a causal relationship cannot be verified, these results strongly support the implication of having an FD. Regarding participants’ number of teeth in this study, in rural areas, those with fewer teeth tended to have an FD. According to the Survey of Dental Diseases in Japan [20], the proportion of those who wear dentures or bridges tends to be higher in rural areas than in urban areas; hence, many denture/bridge-wearers may have come to have an FD for regular oral management.

Conversely, one of the most noteworthy findings of this study is that no association was found between household income and having an FD in urban areas and that this association was only statistically significant among higher-income individuals in intermediate areas. In rural areas, the higher the income, the more likely people are to have an FD. Higher household income is an important factor to consider when implementing regular dental visits, which is a common issue in several countries [21, 22]. Additionally, people living in rural areas tend to have lower incomes than those living in urban areas [23]. Dental care is covered by public healthcare insurance in Japan, and it is thought that barriers to dental visits are lower in Japan than in other countries. Nevertheless, the results of the current study suggest that differences in income by region are associated with having an FD, and a possible reason for this could be the influence of the abovementioned previous reports [21–23].

Our present findings suggest that fewer people have an FD in rural areas, and income inequality is associated with having an FD. Issues related to household income are difficult to resolve individually, and economic policy interventions have been proposed. Therefore, regarding practical implications, our findings may contribute to the planning of oral health policies that consider regional differences. Previous studies have suggested that institutionalizing dental check-ups with reduced out-of-pocket costs would promote dental visits [24, 25]. Particularly in rural areas, such policies must be actively planned to improve access to dental care and disseminated to encourage people to have an FD.

This study has some limitations. First, the study’s participants were randomly sampled from a large population of approximately 1.3 million people registered with the web research company. Although the use of a web-based survey provided the advantage of stratifying participants into equal-size proportions by municipality type, these participants do not represent the entire Japanese population. Thus, a sampling bias for these participants cannot be ruled out. Second, this was a cross-sectional study; therefore, it is not possible to identify a causal relationship between having an FD and each explanatory variable. Third, it has been suggested that dental clinic density may influence the reasons why access to dental services is more of a barrier in rural areas than in urban areas [11]. It has further been reported that regional distribution inequality in the number of dental clinics in Japan is decreasing [26]; however, our current study did not evaluate the association between regional differences in the population with an FD and the distribution of dental clinics. In Japan, when opening a dental clinic, the dentist is free to choose the area in which to practice; therefore, the government has no control over the distribution of dental clinics. Fourth, the participants in this study were between the ages of 20–69 years, and those 70 years or older were not included. Older adults are particularly at risk for oral health problems [27]; therefore, it is important to regularly manage their oral health by having an FD. Further, a report analyzing the relationship between socioeconomic status and oral health among older adults in Japan at different co-payment rates suggests that the inequality in access to dental visits decreases for those aged 75 years and older due to lower co-payment [28]. However, there is a lack of reports assessing the status of people with an FD (especially those over 70 years of age) in Japan regarding regional differences. Further studies should be conducted to determine the factors associated with having an FD.

## Conclusion

This study assessed the status of individuals having an FD among Japanese people by municipality type, classified into three categories—urban, intermediate, and rural areas—using a web-based survey. The proportion of those with an FD was lowest in rural areas (42.3%), followed by intermediate (48.6%) and urban areas (49.7%). Particularly in rural areas, there was a tendency for having an FD among individuals with a higher than lower household income.

These results suggest that rural areas have the fewest people with FD among the three areas, and income inequality is associated with having an FD. Thus, when planning policies designed to encourage people to acquire an FD to manage their oral health, it is necessary to consider regional differences.

### Electronic supplementary material

Below is the link to the electronic supplementary material.

Supplementary Material 1

## Data Availability

The datasets generated and/or analyzed during the current study are not publicly available owing to participant privacy; however, they are available from the corresponding author upon reasonable request.

## Abbreviations

FD: Family dentist
JPY: Japanese Yen
