# Supplementary material for: Urban-rural differences in the prevalence of having a family dentist and their association with income inequality among Japanese individuals: a cross-sectional study
Source: BMC Oral Health. 2024 Jun 27;24:741. doi: 10.1186/s12903-024-04528-8 (PMC11210090; doi:10.1186/s12903-024-04528-8)
Supplement: Supplementary file 1 — Supplementary Material 1 [file 12903_2024_4528_MOESM1_ESM.pdf]

Supplementary Table 1. Characteristics of all study participants in the FD group compared to the non-FD group (Univariate and multivariate modified Poisson regression analysis)

	All study participants	
	Univariate analysis	Multivariate analysis [a]
	PR (95%CI)	PR (95%CI)
Gender		
Men	0.84 (0.77–0.91) ***	0.97 (0.88–1.08)
Women	reference	reference
Age (years)		
20–29	reference	reference
30–39	1.19 (1.02–1.38) *	1.00 (0.87–1.16)
40–49	1.13 (0.97–1.32)	0.94 (0.81–1.08)
50–59	1.27 (1.10–1.46) **	0.99 (0.86–1.14)
60–69	1.49 (1.30–1.71) ***	1.21 (1.05–1.39) **
Household income (million)		
JPY <2	reference	reference
JPY 2–4	1.32 (1.08–1.61) **	1.19 (0.99–1.45)
JPY 4–6	1.33 (1.09–1.62) **	1.19 (0.98–1.44)
JPY 6–8	1.44 (1.18–1.76) ***	1.29 (1.06–1.57) *
JPY ≥8	1.64 (1.36–1.99) ***	1.47 (1.21–1.77) ***
Unknown	1.13 (0.93–1.39)	1.09 (0.90–1.32)
Working status		
Regular worker	reference	reference
Homemaker	1.27 (1.14–1.41) ***	1.06 (0.94–1.21)
Part–time worker	1.04 (0.92–1.16)	1.03 (0.91–1.17)
Unemployed/others	0.80 (0.69–0.93) **	0.92 (0.80–1.07)
Number of teeth		
≥28	0.95 (0.80–1.12)	0.90 (0.78–1.05)
20–27	1.11 (0.94–1.31)	0.95 (0.82–1.11)
≤19	reference	reference
Frequency of brushing teeth		
≥Three times daily	1.53 (1.32–1.77) ***	1.22 (1.06–1.40) **
Twice daily	1.41 (1.22–1.62) ***	1.18 (1.03–1.35) *
Once daily	reference	reference
Sometimes/No brushing	0.48 (0.28–0.83) **	0.56 (0.33–0.95) *
Interdental cleaning		
Yes	2.25 (2.03–2.49) ***	2.06 (1.85–2.28) ***
No	reference	reference
Municipality type		
Urban	1.17 (1.06–1.31) **	
Intermediate	1.50 (1.03–1.28) *	
Rural	reference	

Note: * p<0.05, ** p<0.01, *** p<0.001, FD group = group of those who regularly manage their oral health with a family dentist (FD); PR = prevalence ratio; 95%CI = 95% confidence interval.
a: Multivariate analysis excluding the "Municipality type" variable (Multivariate analysis including the "municipality type" variable was presented in Table 2).

Supplementary Table 2. Characteristics of the study participants in the FD group compared to the non-FD group, classified by municipality type (Univariate modified Poisson regression analysis)

	Urban	Intermediate	Rural
	PR (95%CI)	PR (95%CI)	PR (95%CI)
Gender			
Men	0.97 (0.84–1.11)	0.80 (0.70–0.93) **	0.74 (0.63–0.87) ***
Women	reference	reference	reference
Age (years)			
20–29	reference	reference	reference
30–39	1.07 (0.83–1.37)	1.45 (1.13–1.87) **	1.06 (0.81–1.39)
40–49	1.18 (0.93–1.50)	1.16 (0.88–1.53)	1.05 (0.80–1.38)
50–59	1.14 (0.90–1.46)	1.59 (1.24–2.03) ***	1.09 (0.84–1.43)
60–69	1.59 (1.28–1.97) ***	1.60 (1.26–2.05) ***	1.28 (1.00–1.65)
Household income (million)			
JPY <2	reference	reference	reference
JPY 2–4	0.97 (0.70–1.33)	1.61 (1.13–2.30) **	1.44 (1.01–2.05) *
JPY 4–6	1.06 (0.78–1.44)	1.43 (1.00–2.05)	1.53 (1.08–2.18) *
JPY 6–8	1.26 (0.93–1.71)	1.56 (1.09–2.23) *	1.48 (1.02–2.15) *
JPY ≥8	1.30 (0.97–1.74)	1.80 (1.27–2.56) **	1.83 (1.28–2.62) **
Unknown	1.00 (0.73–1.37)	1.21 (0.84–1.75)	1.20 (0.83–1.73)
Working status			
Regular worker	reference	reference	reference
Homemaker	1.19 (1.00–1.43)	1.24 (1.05–1.47) *	1.40 (1.15–1.70) **
Part-time worker	0.97 (0.79–1.19)	1.06 (0.87–1.28)	1.11 (0.90–1.38)
Unemployed/others	0.93 (0.74–1.16)	0.72 (0.55–0.93) *	0.78 (0.59–1.03)
Number of teeth			
≥28	0.94 (0.71–1.26)	1.08 (0.80–1.46)	0.84 (0.64–1.11)
20–27	1.24 (0.93–1.65)	1.16 (0.86–1.59)	0.93 (0.70–1.24)
≤19	reference	reference	reference
Frequency of brushing teeth			
≥Three times daily	1.41 (1.11–1.79) **	1.31 (1.03–1.67) *	2.01 (1.48–2.72) ***
Twice daily	1.27 (1.00–1.60) *	1.34 (1.07–1.68) *	1.68 (1.25–2.27) **
Once daily	reference	reference	reference
Sometimes/No brushing	0.91 (0.52–1.59)	0.00 (0.00–0.00) ***	0.50 (0.17–1.49)
Interdental cleaning			
Yes	2.27 (1.89–2.72) ***	1.90 (1.62–2.22) ***	2.73 (2.23–3.33) ***
No	reference	reference	reference

Note: * p<0.05, ** p<0.01, *** p<0.001, FD group = group of those who regularly manage their oral health with a family dentist (FD); PR = prevalence ratio; 95%CI = 95% confidence interval.

Supplementary Table 3. Association between tooth number and each variable, classified by age group (Univariate and multivariate negative binomial regression analysis)

	All study participants		20s		30s	
	Univariate analysis	Multivariate analysis	Univariate analysis	Multivariate analysis	Univariate analysis	Multivariate analysis
	PR (95%CI)	PR (95%CI)	PR (95%CI)	PR (95%CI)	PR (95%CI)	PR (95%CI)
Gender						
Men	0.98 (0.96–1.00) *	0.96 (0.94–0.99) **	1.00 (0.96–1.05)	1.00 (0.96–1.05)	0.98 (0.95–1.02)	1.00 (0.96–1.04)
Women	reference	reference	reference	reference	reference	reference
Household income (million)						
JPY <2	reference	reference	reference	reference	reference	reference
JPY 2–4	1.00 (0.96–1.04)	1.00 (0.96–1.04)	0.98 (0.91–1.07)	0.99 (0.91–1.08)	1.06 (0.98–1.15)	1.08 (0.99–1.17)
JPY 4–6	1.03 (0.99–1.07)	1.03 (0.98–1.07)	0.96 (0.88–1.04)	0.97 (0.88–1.06)	1.05 (0.98–1.13)	1.08 (1.00–1.17)
JPY 6–8	1.05 (1.00–1.09) *	1.04 (1.00–1.09)	1.00 (0.91–1.10)	1.01 (0.92–1.11)	1.02 (0.95–1.11)	1.05 (0.96–1.14)
JPY ≥8	1.04 (1.00–1.09) *	1.04 (1.00–1.09)	0.99 (0.90–1.05)	0.99 (0.90–1.06)	1.08 (0.99–1.16)	1.11 (1.02–1.21) *
Unknown	1.03 (0.99–1.07)	1.03 (1.00–1.07)	0.97 (0.90–1.05)	0.98 (0.91–1.06)	1.07 (0.99–1.16)	1.08 (0.99–1.17)
Working status						
Regular worker	reference	reference	reference	reference	reference	reference
Homemaker	1.00 (0.97–1.03)	0.98 (0.95–1.01)	1.02 (0.92–1.14)	1.04 (0.93–1.16)	1.02 (0.97–1.08)	1.02 (0.97–1.09)
Part-time worker	0.98 (0.95–1.01)	0.97 (0.94–1.00) *	0.99 (0.92–1.06)	1.00 (0.92–1.07)	1.04 (0.99–1.09)	1.04 (0.99–1.10)
Unemployed/others	0.99 (0.96–1.02)	0.99 (0.96–1.03)	1.02 (0.97–1.08)	1.02 (0.97–1.09)	1.02 (0.96–1.09)	1.04 (0.97–1.12)
Municipality type						
Urban	1.00 (0.98–1.03)	1.00 (0.98–1.02)	1.02 (0.96–1.07)	1.02 (0.96–1.08)	0.99 (0.95–1.03)	0.99 (0.95–1.04)
Intermediate	1.00 (0.98–1.03)	1.00 (0.97–1.02)	1.02 (0.97–1.08)	1.02 (0.97–1.08)	0.99 (0.95–1.03)	0.99 (0.95–1.04)
Rural	reference	reference	reference	reference	reference	reference
FD						
FD groups	1.00 (0.98–1.02)	1.00 (0.98–1.02)	1.01 (0.97–1.06)	1.01 (0.97–1.06)	0.99 (0.96–1.03)	0.99 (0.96–1.03)
Non-FD groups	reference	reference	reference	reference	reference	reference

Note: * p<0.05, ** p<0.01, *** p<0.001, FD group = group of those who regularly manage their oral health with a family dentist (FD); PR = prevalence ratio; 95%CI = 95% confidence interval.

Supplementary Table 3. (continued)

	40s		50s		60s	
	Univariate analysis	Multivariate analysis	Univariate analysis	Multivariate analysis	Univariate analysis	Multivariate analysis
	PR (95%CI)	PR (95%CI)	PR (95%CI)	PR (95%CI)	PR (95%CI)	PR (95%CI)
Gender						
Men	0.99 (0.95–1.02)	0.99 (0.95–1.04)	0.96 (0.92–1.00)	0.95 (0.89–1.00)	0.96 (0.90–1.03)	0.93 (0.85–1.01)
Women	reference	reference	reference	reference	reference	reference
Household income (million)						
JPY <2	reference	reference	reference	reference	reference	reference
JPY 2–4	1.02 (0.95–1.11)	1.02 (0.94–1.11)	0.93 (0.85–1.01)	0.92 (0.84–1.01)	1.02 (0.91–1.15)	1.01 (0.90–1.14)
JPY 4–6	1.01 (0.94–1.09)	1.01 (0.93–1.09)	1.02 (0.93–1.11)	1.02 (0.93–1.12)	1.07 (0.95–1.21)	1.06 (0.94–1.20)
JPY 6–8	1.03 (0.96–1.12)	1.03 (0.95–1.11)	1.01 (0.93–1.10)	1.01 (0.92–1.11)	1.15 (1.01–1.31) *	1.13 (0.98–1.30)
JPY ≥8	1.01 (0.94–1.09)	1.01 (0.93–1.09)	0.99 (0.91–1.07)	0.99 (0.91–1.08)	1.17 (1.03–1.34) *	1.13 (0.99–1.30)
Unknown	1.03 (0.96–1.12)	1.03 (0.95–1.11)	0.97 (0.89–1.05)	0.95 (0.87–1.04)	1.13 (1.00–1.27) *	1.10 (0.97–1.24)
Working status						
Regular worker	reference	reference	reference	reference	reference	reference
Homemaker	1.02 (0.97–1.08)	1.01 (0.95–1.08)	1.05 (0.98–1.12)	1.01 (0.94–1.09)	1.01 (0.93–1.09)	0.95 (0.85–1.06)
Part-time worker	1.00 (0.95–1.04)	0.98 (0.93–1.04)	1.02 (0.96–1.08)	0.99 (0.92–1.06)	0.88 (0.80–0.97) **	0.86 (0.77–0.96) **
Unemployed/others	1.00 (0.94–1.06)	0.99 (0.93–1.06)	1.00 (0.92–1.09)	0.99 (0.91–1.08)	0.95 (0.87–1.04)	0.97 (0.88–1.06)
Municipality type						
Urban	1.00 (0.96–1.04)	0.99 (0.95–1.04)	1.00 (0.95–1.07)	1.01 (0.95–1.06)	1.01 (0.93–1.09)	0.98 (0.91–1.06)
Intermediate	0.99 (0.95–1.03)	0.99 (0.95–1.03)	1.01 (0.96–1.07)	1.01 (0.96–1.07)	0.99 (0.92–1.08)	0.97 (0.90–1.05)
Rural	reference	reference	reference	reference	reference	reference
FD						
FD groups	1.02 (0.98–1.06)	1.02 (0.98–1.06)	0.98 (0.94–1.03)	0.98 (0.93–1.02)	1.06 (0.99–1.13)	1.05 (0.99–1.13)
Non-FD groups	reference	reference	reference	reference	reference	reference

Note: * p<0.05, ** p<0.01, *** p<0.001, FD group = group of those who regularly manage their oral health with a family dentist (FD); PR = prevalence ratio; 95%CI = 95% confidence interval.

Supplementary Table 4. Association between frequency of brushing teeth and each variable, classified by age group (Univariate and multivariate negative binomial regression analysis)

	All study participants		20s		30s	
	Univariate analysis	Multivariate analysis	Univariate analysis	Multivariate analysis	Univariate analysis	Multivariate analysis
	PR (95%CI)	PR (95%CI)	PR (95%CI)	PR (95%CI)	PR (95%CI)	PR (95%CI)
Gender						
Men	0.87 (0.83–0.92) ***	0.87 (0.81–0.93) ***	0.87 (0.77–0.99) *	0.87 (0.76–1.00) *	0.89 (0.78–1.00)	0.85 (0.74–0.98) *
Women	reference	reference	reference	reference	reference	reference
Household income (million)						
JPY <2	reference	reference	reference	reference	reference	reference
JPY 2–4	1.04 (0.93–1.17)	1.01 (0.90–1.13)	1.15 (0.91–1.46)	1.08 (0.84–1.39)	1.04 (0.79–1.38)	1.01 (0.75–1.35)
JPY 4–6	1.06 (0.95–1.19)	1.03 (0.92–1.16)	1.15 (0.90–1.47)	1.09 (0.83–1.42)	1.06 (0.82–1.38)	1.03 (0.78–1.37)
JPY 6–8	1.06 (0.95–1.20)	1.03 (0.91–1.17)	1.14 (0.87–1.49)	1.06 (0.80–1.41)	1.06 (0.80–1.39)	1.02 (0.76–1.37)
JPY ≥8	1.11 (0.99–1.24)	1.01 (0.94–1.20)	1.21 (0.92–1.58)	1.11 (0.83–1.49)	1.12 (0.85–1.48)	1.07 (0.79–1.45)
Unknown	1.05 (0.94–1.17)	1.02 (0.91–1.14)	1.04 (0.83–1.32)	1.02 (0.80–1.29)	1.07 (0.81–1.42)	1.03 (0.77–1.37)
Working status						
Regular worker	reference	reference	reference	reference	reference	reference
Homemaker	1.08 (0.99–1.17)	0.98 (0.89–1.07)	1.02 (0.76–1.37)	0.96 (0.71–1.30)	1.00 (0.83–1.20)	0.89 (0.72–1.09)
Part-time worker	1.03 (0.96–1.12)	0.97 (0.89–1.06)	0.92 (0.75–1.13)	0.93 (0.75–1.14)	1.00 (0.83–1.18)	0.94 (0.77–1.14)
Unemployed/others	0.93 (0.85–1.01)	0.94 (0.86–1.03)	0.86 (0.74–1.00)	0.92 (0.77–1.09)	0.93 (0.74–1.16)	0.93 (0.72–1.19)
Municipality type						
Urban	1.00 (0.94–1.07)	0.99 (0.93–1.06)	1.00 (0.85–1.16)	0.98 (0.84–1.15)	0.94 (0.81–1.09)	0.92 (0.79–1.07)
Intermediate	1.00 (0.93–1.07)	0.99 (0.92–1.06)	0.99 (0.85–1.15)	0.99 (0.85–1.16)	0.96 (0.83–1.12)	0.95 (0.82–1.11)
Rural	reference	reference	reference	reference	reference	reference
FD						
FD groups	1.12 (1.06–1.18) ***	1.10 (1.04–1.16) **	1.14 (1.01–1.30) *	1.14 (0.98–1.27)	1.10 (0.97–1.24)	1.10 (0.95–1.23)
Non-FD groups	reference	reference	reference	reference	reference	reference

Note: * p<0.05, ** p<0.01, *** p<0.001, FD group = group of those who regularly manage their oral health with a family dentist (FD); PR = prevalence ratio; 95%CI = 95% confidence interval.

Supplementary Table 4. (continued)

	40s		50s		60s	
	Univariate analysis	Multivariate analysis	Univariate analysis	Multivariate analysis	Univariate analysis	Multivariate analysis
	PR (95%CI)	PR (95%CI)	PR (95%CI)	PR (95%CI)	PR (95%CI)	PR (95%CI)
Gender						
Men	0.88 (0.78–1.00) *	0.86 (0.73–1.01)	0.85 (0.76–0.97) *	0.86 (0.73–1.02)	0.87 (0.77–0.99) *	0.92 (0.77–1.10)
Women	reference	reference	reference	reference	reference	reference
Household income (million)						
JPY <2	reference	reference	reference	reference	reference	reference
JPY 2–4	0.95 (0.72–1.27)	0.94 (0.70–1.27)	1.04 (0.81–1.32)	0.99 (0.77–1.27)	1.02 (0.81–1.28)	1.00 (0.79–1.26)
JPY 4–6	0.98 (0.74–1.30)	0.98 (0.73–1.31)	1.03 (0.80–1.32)	1.00 (0.76–1.30)	1.06 (0.84–1.34)	1.05 (0.82–1.33)
JPY 6–8	1.04 (0.79–1.37)	1.03 (0.77–1.37)	1.10 (0.85–1.41)	1.08 (0.83–1.41)	0.97 (0.74–1.25)	0.96 (0.73–1.26)
JPY ≥8	1.06 (0.81–1.39)	1.05 (0.79–1.41)	1.12 (0.89–1.39)	1.08 (0.85–1.37)	0.99 (0.76–1.28)	0.98 (0.75–1.29)
Unknown	1.01 (0.76–1.32)	0.98 (0.74–1.29)	1.11 (0.88–1.41)	1.08 (0.84–1.37)	1.02 (0.81–1.28)	1.00 (0.79–1.26)
Working status						
Regular worker	reference	reference	reference	reference	reference	reference
Homemaker	1.06 (0.88–1.27)	0.94 (0.76–1.17)	1.15 (0.97–1.35)	1.02 (0.83–1.25)	1.16 (0.99–1.36)	1.07 (0.86–1.32)
Part-time worker	1.05 (0.90–1.23)	0.95 (0.79–1.15)	1.09 (0.93–1.27)	0.98 (0.81–1.20)	1.12 (0.93–1.35)	1.06 (0.85–1.31)
Unemployed/others	0.97 (0.76–1.22)	0.96 (0.74–1.24)	0.92 (0.73–1.17)	0.94 (0.73–1.21)	1.03 (0.86–1.22)	1.01 (0.84–1.21)
Municipality type						
Urban	1.00 (0.86–1.17)	0.98 (0.84–1.14)	1.04 (0.90–1.21)	1.03 (0.88–1.20)	1.04 (0.90–1.22)	1.03 (0.88–1.20)
Intermediate	1.00 (0.86–1.16)	0.99 (0.85–1.15)	1.03 (0.89–1.20)	1.01 (0.86–1.17)	0.99 (0.85–1.16)	0.99 (0.85–1.15)
Rural	reference	reference	reference	reference	reference	reference
FD						
FD groups	1.12 (0.99–1.27)	1.10 (0.97–1.25)	1.11 (0.98–1.25)	1.09 (0.96–1.23)	1.10 (0.97–1.25)	1.08 (0.95–1.23)
Non-FD groups	reference	reference	reference	reference	reference	reference

Note: * $p<0.05$, ** $p<0.01$, *** $p<0.001$, FD group = group of those who regularly manage their oral health with a family dentist (FD); PR = prevalence ratio; 95%CI = 95% confidence interval.

Supplementary Table 5. Association between interdental cleaning and each variable, classified by age group (Univariate and multivariate modified Poisson regression analysis)

	All study participants		20s		30s	
	Univariate analysis	Multivariate analysis	Univariate analysis	Multivariate analysis	Univariate analysis	Multivariate analysis
	PR (95%CI)	PR (95%CI)	PR (95%CI)	PR (95%CI)	PR (95%CI)	PR (95%CI)
Gender						
Men	0.75 (0.70–0.81) ***	0.80 (0.73–0.87) ***	0.78 (0.62–0.97) *	0.78 (0.63–0.98) *	0.85 (0.72–1.00)	0.90 (0.75–1.08)
Women	reference	reference	reference	reference	reference	reference
Household income (million)						
JPY <2	reference	reference	reference	reference	reference	reference
JPY 2–4	1.17 (0.99–1.38)	1.05 (0.90–1.23)	1.31 (0.79–2.17)	0.93 (0.57–1.53)	1.14 (0.74–1.76)	1.13 (0.75–1.70)
JPY 4–6	1.25 (1.07–1.47) **	1.14 (0.97–1.33)	1.80 (1.11–2.93) *	1.26 (0.77–2.06)	1.33 (0.90–1.98)	1.27 (0.87–1.87)
JPY 6–8	1.30 (1.11–1.54) **	1.16 (0.99–1.36)	2.09 (1.28–3.41) **	1.46 (0.89–2.39)	1.35 (0.89–2.04)	1.23 (0.83–1.81)
JPY ≥8	1.30 (1.11–1.53) **	1.10 (0.94–1.29)	1.87 (1.12–3.11) *	1.18 (0.70–1.99)	1.43 (0.95–2.16)	1.24 (0.83–1.86)
Unknown	1.06 (0.90–1.25)	0.98 (0.84–1.15)	1.11 (0.66–1.84)	1.00 (0.61–1.63)	1.08 (0.70–1.68)	1.01 (0.68–1.50)
Working status						
Regular worker	reference	reference	reference	reference	reference	reference
Homemaker	1.36 (1.25–1.48) ***	1.12 (1.01–1.23) *	0.92 (0.57–1.49)	0.92 (0.58–1.44)	1.25 (1.03–1.51) *	1.12 (0.89–1.39)
Part-time worker	1.04 (0.94–1.16)	0.95 (0.86–1.06)	0.61 (0.40–0.93) *	0.68 (0.45–1.01)	0.88 (0.68–1.14)	0.97 (0.75–1.27)
Unemployed/others	0.84 (0.74–0.96) **	0.93 (0.82–1.06)	0.56 (0.41–0.77) ***	0.68 (0.48–0.95) *	0.76 (0.53–1.09)	0.99 (0.67–1.45)
Municipality type						
Urban	1.12 (1.02–1.22) *	1.07 (0.98–1.16)	1.16 (0.90–1.51)	1.13 (0.88–1.46)	1.17 (0.96–1.43)	1.14 (0.94–1.38)
Intermediate	1.00 (0.91–1.10)	0.96 (0.88–1.04)	0.90 (0.67–1.20)	0.92 (0.70–1.20)	1.14 (0.93–1.41)	1.06 (0.87–1.29)
Rural	reference	reference	reference	reference	reference	reference
FD						
FD groups	1.92 (1.77–2.08) ***	1.84 (1.70–1.99) ***	2.16 (1.73–2.70) ***	1.97 (1.57–2.45) ***	1.91 (1.61–2.27) ***	1.85 (1.55–2.21) ***
Non-FD groups	reference	reference	reference	reference	reference	reference

Note: * $p<0.05$, ** $p<0.01$, *** $p<0.001$, FD group = group of those who regularly manage their oral health with a family dentist (FD); PR = prevalence ratio; 95%CI = 95% confidence interval.

Supplementary Table 5. (continued)

	40s		50s		60s	
	Univariate analysis	Multivariate analysis	Univariate analysis	Multivariate analysis	Univariate analysis	Multivariate analysis
	PR (95%CI)	PR (95%CI)	PR (95%CI)	PR (95%CI)	PR (95%CI)	PR (95%CI)
Gender						
Men	0.73 (0.62–0.87) ***	0.74 (0.62–0.89) **	0.67 (0.57–0.77) ***	0.76 (0.63–0.93) **	0.75 (0.65–0.87) ***	0.78 (0.64–0.95) *
Women	reference	reference	reference	reference	reference	reference
Household income (million)						
JPY <2	reference	reference	reference	reference	reference	reference
JPY 2–4	1.06 (0.72–1.58)	1.10 (0.75–1.62)	1.34 (0.99–1.81)	1.22 (0.91–1.63)	1.08 (0.83–1.41)	0.97 (0.74–1.26)
JPY 4–6	1.07 (0.72–1.58)	1.09 (0.73–1.61)	1.24 (0.90–1.71)	1.20 (0.88–1.64)	1.10 (0.83–1.44)	1.02 (0.78–1.33)
JPY 6–8	1.23 (0.84–1.78)	1.19 (0.82–1.72)	1.18 (0.85–1.63)	1.20 (0.87–1.64)	1.05 (0.77–1.42)	0.94 (0.70–1.26)
JPY ≥8	1.09 (0.74–1.59)	1.04 (0.71–1.52)	1.23 (0.92–1.66)	1.16 (0.86–1.55)	1.11 (0.83–1.49)	0.95 (0.71–1.27)
Unknown	1.08 (0.74–1.59)	1.00 (0.69–1.43)	1.15 (0.84–1.57)	1.13 (0.84–1.53)	0.97 (0.74–1.28)	0.88 (0.67–1.16)
Working status						
Regular worker	reference	reference	reference	reference	reference	reference
Homemaker	1.30 (1.05–1.60) *	1.00 (0.80–1.26)	1.46 (1.25–1.72) ***	1.19 (0.98–1.44)	1.34 (1.14–1.59) **	1.03 (0.82–1.28)
Part-time worker	1.08 (0.87–1.33)	0.91 (0.73–1.12)	1.39 (1.18–1.63) ***	1.14 (0.94–1.38)	0.99 (0.78–1.26)	0.81 (0.63–1.05)
Unemployed/others	1.29 (1.01–1.66) *	1.31 (1.02–1.68) *	0.87 (0.62–1.22)	0.98 (0.70–1.36)	1.03 (0.84–1.27)	1.00 (0.81–1.22)
Municipality type						
Urban	1.13 (0.93–1.36)	1.08 (0.90–1.29)	1.07 (0.90–1.27)	1.04 (0.89–1.22)	1.08 (0.91–1.28)	0.98 (0.84–1.14)
Intermediate	0.97 (0.78–1.19)	0.95 (0.79–1.16)	1.00 (0.84–1.20)	0.92 (0.78–1.09)	0.98 (0.82–1.17)	0.93 (0.78–1.10)
Rural	reference	reference	reference	reference	reference	reference
FD						
FD groups	1.94 (1.64–2.29) ***	1.87 (1.58–2.21) ***	1.62 (1.40–1.89) ***	1.56 (1.35–1.81) ***	1.89 (1.58–2.27) ***	1.85 (1.54–2.21) ***
Non-FD groups	reference	reference	reference	reference	reference	reference

Note: * p<0.05, ** p<0.01, *** p<0.001, FD group = group of those who regularly manage their oral health with a family dentist (FD); PR = prevalence ratio; 95%CI = 95% confidence interval.
